# Osmotic Dehydration, Drying Kinetics, and Quality Attributes of Osmotic Hot Air-Dried Mango as Affected by Initial Frozen Storage

**DOI:** 10.3390/foods11030489

**Published:** 2022-02-08

**Authors:** Pramote Khuwijitjaru, Supawadee Somkane, Kyuya Nakagawa, Busarakorn Mahayothee

**Affiliations:** 1 Department of Food Technology, Faculty of Engineering and Industrial Technology, Silpakorn University, Nakhon Pathom 73000, Thailand; khuwijitjaru_p@su.ac.th (P.K.); supawadee.oil15@gmail.com (S.S.); 2 Department of Chemical Engineering, Faculty of Engineering, Kyoto University, Katsura, Nishikyo-ku, Kyoto 615-8510, Japan; kyuya@cheme.kyoto-u.ac.jp

**Keywords:** mango (*Mangifera indica*), freezing, osmotic dehydration, hot air drying, quality

## Abstract

Using frozen mango for osmotic hot air drying is still uncommon due to a lack of knowledge on the effect of the freezing process on the final product’s quality attributes. This study aimed to investigate the effect of the freezing method (slow and quick freezing) and frozen storage time at −18 °C (0, 1, and 2 months) on mass transfer kinetics during osmotic dehydration, drying kinetics during hot air drying, and final quality attributes of the dried mango. The results indicated that Peleg’s model could describe the water loss and solid gain during the osmotic dehydration in a 38° Brix sugar solution. Freezing before osmotic dehydration reduced the water loss rate while increasing the solid uptake content. Frozen mangoes showed slightly higher drying rates at 50 and 60 °C than the fresh ones. Freezing and frozen storage also retarded the browning reaction and polyphenol oxidase activities. The osmotic-dried mango obtained from frozen mangoes showed a chewy and gummy texture, which could be considered a distinctive texture characteristic for dried mango.

## 1. Introduction

Mango (*Mangifera indica*) is a well-known tropical fruit with a total world production of 55 million tons in 2020 [1]. Mangoes are also processed into various products such as packaged or frozen juice and puree, pickled mango, and dried mango [2] and therefore contribute to an economy in many countries.

Drying of fruit is a simple preservation process that has been widely practiced [3]. Mango can be directly dried without pretreatment or with osmotic dehydration pretreatment using sugar solution before hot air drying. However, because mango is a seasonal fruit and the high price but low quality of the off-season mango, manufacturers usually store cut mango pieces by submerging them in a solution of calcium chloride, citric acid, and sulfiting agent at room temperature [4] to preserve mangoes for processing year-round. Despite sulfite’s effectiveness as a preservative and antibrowning agent, it can cause undesirable health effects to humans, and thus it is listed as a food allergen in many countries [5]. In this context, an alternative method for mango storage before osmotic hot air drying during the off-season is needed. Currently, freezing is a very common and affordable process for the food industry. This technology has been used to preserve fruits for off-season usage with higher quality compared to other methods. Most chemical, biochemical, and microbial changes are effectively retarded during freezing, while sensorial changes are also minimized. However, freezing can adversely affect the cell structure of mango [6,7] and may result in poor quality of dried mango. Additionally, since freezing does not completely inhibit the undesirable enzymatic activities, particularly polyphenol oxidases, which catalyze the undesirable browning reaction in mango [8], it may affect the quality attributes of the final product.

The effect of freezing as a pretreatment for the osmotic dehydration process of plant tissues has been studied. Kowalska, et al. [9] reported that freezing resulted in lower water loss and higher solid gain in pumpkin slices during the osmotic dehydration in sucrose solution, which was also in agreement with the result for apple slices [10]. In addition, the effect of freezing on the quality of dried products has been reported for some fruits and vegetables such as pumpkin [11], beetroot [12], and cranberries [13]. Generally, the formation of ice crystals increases the porosity of structure and therefore enhances moisture removal during drying. Li et al. [14] reported an approximately 5–13% drip loss in mango after frozen storage at different temperatures for 6 months. However, a study on the effect of freezing and frozen storage on both mass transfer kinetics during the osmotic dehydration and drying kinetics during the hot air dying of mango has never been published. Furthermore, the quality of osmotic hot air-dried mango from frozen mango has never been reported. Due to this lack of knowledge, using frozen mango for osmotic hot air drying has not been industrially employed. In this study, therefore, the effects of the freezing method and frozen storage time on the kinetics of osmotic dehydration and kinetics of hot air drying of mango were investigated. In addition, the quality attributes of dried mango obtained from these treatments were also determined to evaluate the feasibility of freezing as an alternative method for mango storage for off-season use.

## 2. Materials and Methods

### 2.1. Mango Preparation

Mango cv. Kaew Kamin, which is gaining popularity as a raw material for producing dehydrated mango in Thailand, was used in this study. Mature green mangoes were purchased and ripened at room temperature until the total soluble solids content (TSS) reached 18.6–19.6° Brix, which provided a suitable firmness (Table S1). Ripe mangoes were cut into rectangular slabs (40 mm × 60 mm × 10 mm) and immersed in 1% calcium chloride solution containing 1% citric acid (pH 2.50 ± 0.18) for 3 h. After that, the samples were frozen using a conventional freezer (SF-PC697, Panasonic, Chachoengsao, Thailand) (−18 °C, slow freezing) and an air-blast freezer (GCM015S, Hiber, Milan, Italy) (−40 °C, quick freezing) to reach the temperature in the geometric center of the fruit of −18 °C, which took 6 and 1.17 h, respectively. The temperatures were measured using type-K thermocouples connected to a data logger (GL220, Graphtec, Yokohama, Japan) (Figure S1). Frozen samples were stored at −18 °C for 1 and 2 months for further studies.

### 2.2. Osmotic Dehydration

Fresh and frozen mango slabs after 0, 1, and 2 months of storage in their frozen state were soaked in osmotic solution (38° Brix) containing sucrose and fructose (ratio of 8:2 *w*/*w*), 0.15% (*w*/*v*) ascorbic acid, and 0.2% (*w*/*v*) citric acid. The osmotic solution compositions were from our preliminary study, which provided a mildly sweet final product after hot air drying. Each mango slab (30 g) was individually soaked in 60 mL of the solution at room temperature without mechanical agitation. For frozen samples, the temperature of the osmotic solution was approximately 19 °C at the beginning and reached room temperature (31 °C) at the end of the experiment. Every 2 h, two samples were taken and placed on a wire rack for 5 min each side to remove excess solution from the surface. Then, the samples were weighed and measured for TSS and moisture content. Osmotic dehydration experiments were conducted in duplicate.

Normalized water content (NWC) and normalized solid content (NSC) were calculated using Equations (1) and (2) [9]. NWC and NSC were in units of g/g initial water content (i.w.c.) and g/g initial dry matter (i.d.m.), respectively.

(1) $$\mathrm{NWC}\;\left(g/g\;i.w.c.\right)=\frac{\left(1-s_{t}\right)\;\cdot \;m_{t}}{\left(1-s_{0}\right)\;\cdot \;m_{0}}$$

(2) $$\mathrm{NSC}\;\left(g/g\;i.d.m.\right)=\frac{s_{t}\;\cdot \;m_{t}}{s_{0}\;\cdot \;m_{0}}$$

where *s*_0_, *m*_0_, *s_t_*, and *m_t_* are solid content (g/g sample) and sample mass (g) at time 0 and *t* h, respectively.

An empirical model introduced by Peleg [15] (Equation (3)) was used to describe the changes of NWC and NSC with time.

(3) $$Y\;={\;Y}_{0}\;\pm \;\frac{t}{k_{1}+k_{2}\;\cdot \;t}$$

where Y_0_ and Y represent NWC or NSC at time 0 and *t* h, respectively, and *k*_1_ and *k*_2_ are empirical model parameters called Peleg’s rate constant and Peleg’s capacity constant, respectively. R software [16] was used to estimate the values of *k*_1_ and *k*_2_ with their standard errors (s.e.) using a nonlinear regression method. The residual standard error (RSE) and the coefficient of determination (R^2^) were used to assess how well the models fitted the data:

(4) $$\mathrm{RSE}\;=\sqrt{\frac{\sum_{i=1}^{n}{\left(y_{i}-{\hat{y}}_{i}\right)}^{2}}{n-k}}$$

where$\;y_{i}$, ${\hat{y}}_{i}$, *n*, and *k* represent observation value, predicted value, number of observations, and number of parameters in the model, respectively.

(5) $$R^{2}=1-\frac{\sum_{i=1}^{n}{\left(y_{i}-{\hat{y}}_{i}\right)}^{2}}{\sum_{i=1}^{n}{\left(y_{i}-\bar{y}\right)}^{2}}$$

where $\bar{y}$ represents mean of observation values.

The value of NWC or NSC at equilibrium, Y*_e_* was obtained from Equation (6):

(6) $$Y_{e}={\;Y}_{0}\;\pm \;\frac{1}{k_{2}}$$

### 2.3. Hot Air Drying

The osmotically dehydrated mangoes were dried at 50 and 60 °C using a hot air tray dryer (Kluay Nam Thai Trading Group, Bangkok, Thailand) with an average air velocity above the tray of 0.23 m/s (Testo 405-V1 thermal anemometer, Testo, Titisee-Neustadt, Germany). Eight mango slabs (approximately 200 g) were dried on a perforated tray and weighed every 2 h until reaching a constant weight. The Page model [17], which is relatively simple and has been successfully used for thin-layer drying kinetics of several fruits and vegetables [18], was used to describe the drying behavior of mango slabs. The model is shown in Equation (7):

(7) $$\mathrm{MR}\;=\frac{M_{t}-M_{e}}{M_{0}-M_{e}}=\;\exp\left(-k\;\cdot {\;t}^{n}\right)$$

where MR is moisture ratio, *M*_0_, *M_t_*, and *M_e_* are moisture content (dry basis, d.b.) at time 0, t min, and at equilibrium, respectively, *k* and *n* are the model parameters. The final moisture content was considered as *M_e_*. R software was used to perform nonlinear regression to estimate the values of *k* and *n* with their standard errors. RSE and R^2^ were used to assess how well the models fitted the data.

Drying rate (g water/g dry solid/h/m^2^) at specific time intervals was calculated using Equation (8):

(8) $$\mathrm{Drying}\;\mathrm{rate}=\frac{X_{1}-X_{2}}{t_{2}-t_{1}}\times \frac{1}{A}$$

where *X*_1_ and *X*_2_ are moisture contents (g water/g dry solid) at different times *t*_1_ and *t*_2_ (h), respectively, and *A* is the surface area of the sample (m^2^). Hot air drying experiments were conducted in duplicate.

### 2.4. Effect of Frozen Storage on Osmotic Hot Air-Dried Mango Quality Attributes

Frozen mangoes, which were stored at −18 °C for 1 and 2 months, were osmotic dehydrated and hot air dried at 60 °C. The dried mango samples were measured for their quality attributes, including moisture content, water activity, color parameters, browning index, polyphenol oxidase activity, total phenolic content, and textural properties.

### 2.5. Analysis Methods

#### 2.5.1. Moisture Content, Water Activity, Total Soluble Solids, and Titratable Acidity

Moisture content was determined using an oven drying method at 105 °C to a constant weight according to AOAC [19]. Water activity was determined using a water activity meter (AQUALAB 4TE, METER group, Pullman, WA, USA). TSS was measured using a hand-held refractometer (PAL-1, ATAGO, Tokyo, Japan). Titratable acidity was measured by 0.1 N NaOH titration using phenolphthalein as an indicator [19].

#### 2.5.2. Color Parameters

The color parameters of the samples were measured using a color spectrophotometer (Colorflex EZ, Hunter Associates Laboratory, Reston, VA, USA) as CIE L* (lightness), a* (redness/greenness), b* (yellowness/blueness). Total color difference (ΔE*) were calculated using the following formulas:

(9) $$\Delta E^{*}\;=\sqrt{\Delta {L^{*}}^{2}+\Delta {a^{*}}^{2}+\Delta {b^{*}}^{2}}\;$$

where Δ refers to the difference between dried mangoes obtained from fresh and frozen mangoes.

#### 2.5.3. Browning Index

Browning index was measured to represent brown pigments formed in mango samples according to a method described by Baloch, et al. [20] with some modifications. The mango sample (3 g) was soaked in 2% acetic acid for 10 min and homogenized with a rotor-stator homogenizer (UltraTurrax T25 Basic, IKA, Staufen, Germany). The mixture was centrifuged at 8000 rpm for 15 min at room temperature, and the supernatant was collected. Absorbance of the supernatant at 420 nm (OD) from a UV-vis spectrophotometer (Genesys 10S, Thermo Scientific, Waltham, MA, USA) was used to calculate the browning index (OD/g d.m.).

#### 2.5.4. Polyphenol Oxidase Activity

Polyphenol oxidase (PPO) activity was determined according to the method from Banerjee, et al. [21]. The mango sample (5 g) was homogenized with 15 mL of 0.2 M phosphate buffer (pH 6.8) for 30 s. The mixture was centrifuged at 12,000 rpm for 20 min at 4 °C. The supernatant (0.5 mL) was mixed with 0.2 M phosphate buffer (pH 6.8) (2.5 mL) and 1 M catechol (0.5 mL), and the mixture was incubated at 30 °C for 5 min. Absorbance at 420 nm was measured using a spectrophotometer (Genesys 10S). A unit of PPO activity (U) was defined as an increase of 0.01 unit of absorbance in 1 min. The PPO activity of mango was reported as U/g d.m.

#### 2.5.5. Total Phenolic Content

Total phenolic content (TPC) was measured using Folin–Ciocalteu’s reagent [22]. The mango sample (3 g fresh sample or 1 g dry sample) was mixed with 20 mL of 80% methanol in a centrifuge tube using a vortex mixer for 15 s. Then, the tube was placed in an ultrasonic bath (360D, Advance Ceramics Technology, Pulau Pinang, Malaysia) for 30 min to accelerate the extraction. The extract was collected after filtration with Whatman No. 4 paper. The residue was re-extracted twice using 15 and 10 mL of 80% methanol, respectively. The pooled extract was adjusted to 50 mL with 80% methanol. An aliquot of the sample (0.1 mL) was mixed with 10% Folin–Ciocalteu’s reagent (1 mL). After 5 min, 7.5% sodium carbonate (1.6 mL) was added to the mixture. The mixture was mixed and kept in the dark for 120 min before being measured for absorbance at 765 nm using a spectrophotometer (Genesys 10S). Gallic acid solutions (0–100 mg/L) were used to prepare a calibration curve. TPC was expressed as mg gallic acid equivalent (GAE)/g d.m.

#### 2.5.6. Textural Properties

Texture properties of the dried mango were evaluated using a Texture Analyzer (TA.XTPlus, Stable Micro Systems, Surrey, UK) equipped with a 2 mm cylindrical stainless steel probe (p/2). Two compression cycles were performed using a pre-test speed of 1 mm/s, test speed of 1 mm/s, post-test speed of 10 mm/s, and compression distance of 2 mm. Hardness, adhesiveness, springiness, cohesiveness, gumminess, and chewiness were evaluated in the same way as those in the texture profile analysis (TPA) method [23].

### 2.6. Statistical Analysis

All osmotic dehydration and hot air drying experiments were performed in duplicate. Analysis of variance (ANOVA) was used to identify the significance of treatment on quality attributes of the dried mangoes. Duncan’s multiple range test was used for multiple means comparison. Multiple comparison test of the kinetic parameters from Peleg’s model or Page’s model was performed with Holm’s procedure at a significant level of 0.05 using the *aomisc* package in R software [24].

## 3. Results and Discussion

### 3.1. Osmotic Dehydration Kinetics

**Figure 1:**
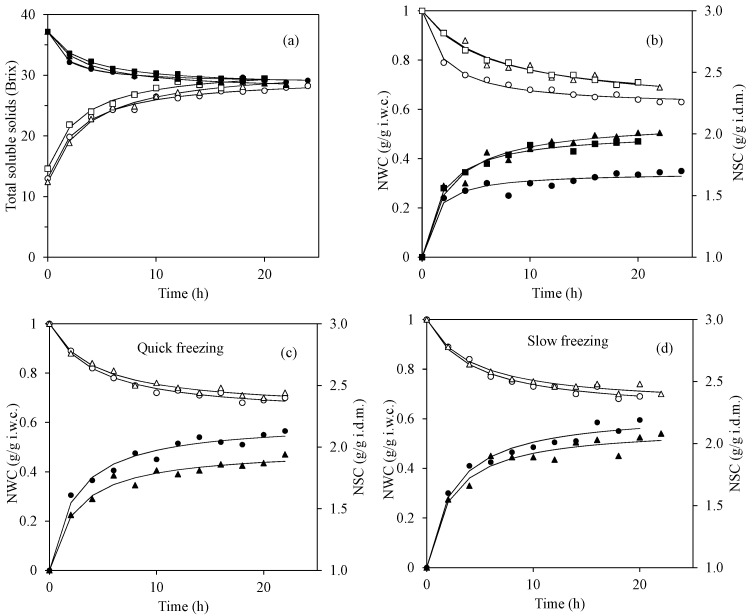
Changes in (**a**) total soluble solids (TSS) content of mango slabs (open symbols) and osmotic solution (closed symbols) and (**b**) NWC (open symbols) and NSC (closed symbols) during the osmotic dehydration of fresh (○, ●), quick frozen (△, ▲), and slow-frozen (□, ■) mango slabs. Changes in NWC (open symbols) and NSC (closed symbols) during the osmotic dehydration of (**c**) quick frozen and (**d**) slow frozen for 1 (○, ●) and 2 month (△, ▲). Lines were drawn using Peleg’s model.

The mango samples used in this study were ripe mangoes with physical and chemical properties shown in Table S1. The mango slabs were pretreated with the solution containing calcium chloride and citric acid before freezing. Calcium is known to improve the texture of fruit by interacting with pectin in fruit structure to form calcium pectate, which is not soluble in water and therefore helps increase the firmness of frozen fruit [25]. The addition of citric acid prevents both enzymatic and non-enzymatic browning reactions by lowering the pH. The optimal pH for polyphenol oxidase (PPO), which catalyzes the oxidation of phenolic compounds and leads to brown pigment formation, is approximately 6.0 [26,27]. In addition, citric acid also chelates copper in the active site of PPO and thus inhibits the activity [28]. Tangtua, Leksawasdi, and Rattanapanone [8] reported about a 64% reduction in PPO activity in mango by soaking with 1% citric mixed with 1% CaCl_2_ solution. Lowering pH also prevents Maillard reaction during the hot air drying and storage [29]. As shown in Figure 1a, the initial TSS of mango slabs after the pretreatment with calcium chloride and citric acid solution was lowered to approximately 13–15° Brix. This was because the pretreatment solution was hypotonic, and therefore, the mango slabs gained water and also lost some sugar. After the osmotic dehydration in the sugar solution with approximately 38° Brix, the TSS of mango slabs increased rapidly after 2–4 h and then gradually increased and reached a plateau at approximately 28–29° Brix, which were in equilibrium with the decrease in TSS in the solution. The times required to reach the equilibrium TSS were 24, 20, and 16 h for fresh, quick-frozen, and slow-frozen mangoes, respectively. In addition, after the frozen storage for 1 and 2 months, the time required to reach the equilibrium TSS was further shortened to 14 h for all treatments (data not shown).

As shown in Figure 1b–d, Peleg’s model could well describe the changes in water content (NWC) and solid content (NSC) during the osmotic dehydration of mango slabs. Table 1 shows that the models provided low RSE (0.01–0.06) and high *R*^2^ (0.9497–0.9943) values. The model has been frequently used to describe the changes in the amount of water and solid during osmotic dehydration using several types of osmotic solutions [30,31,32].

Peleg’s rate constant (*k*_1_) can be used to describe the mass transfer rates of water and solid between the osmotic solution and the mango pieces. The estimated *k*_1_ values (Table 1) clearly showed that the fresh mango slabs lost their water at a higher rate than both quick-frozen and slow-frozen mangoes, but the normalized water content at equilibrium (NWC_e_), which was calculated from the *k*_2_, revealed that all samples contained similar water content at approximately 0.6 g/g i.w.c. (Figure 1b). This means that approximately 40% of water in the mango sample was removed during the dehydration process. In contrast, the frozen mangoes gained solid contents from the osmotic solution at significantly higher rates than the fresh one, and the equilibrium normalized solid contents (NSC_e_) (2.02–2.11 g/g i.d.m.) were also approximately 20–25% higher than that of the fresh mangoes (1.69 g/g i.d.m.). These results reflected the fact that freezing caused damage to the mango cell structure. The formation of ice crystals can result in both physical (cell rupture by ice crystal growth) and chemical damages (biochemical reactions after cell fracture) [33]. Eventually, freezing of mango tissues resulted in a more open structure after thawing in the osmotic solution and therefore promoted the migration of sugars from the osmotic solution into the sample while water as ice in the mango pieces moved slowly from the cell toward the solution, especially at the beginning of the osmotic dehydration process [9]. Kowalska, Lenart, and Leszczyk [9] also reported that raw pumpkin lost its water easier than the frozen one in sucrose solution, and the solid content in the frozen pumpkin was around three times higher than that of fresh pumpkin. In addition, Taiwo, Angersbach, Ade-Omowaye, and Knorr [10] found that freezing increased both water loss and solid gain of strawberry halves during the osmotic dehydration in 50° Brix solution. In this study, however, the difference in water loss and solid gain between quick- and slow-freezing processes (0 month) was not significant. This suggested that the slow and quick freezing provided not much different effects on the mango cell structure when both freezing processes were stopped at −18 °C.

After 1 and 2 months of frozen storage at −18 °C (Figure 1c,d), *k*_1_ and *k*_2_ for NWC (Table 1) did not change significantly (*p* > 0.05) from those after immediate freezing (0 months). In addition, for NSC, *k*_1_ values were also similar for all samples, but there were significant differences for *k*_2_ values. However, the differences were small, and there were no specific trends. In addition, the NWC_e_ and NSC_e_ did not significantly change during the 2 months of frozen storage. This suggested that for a relatively short time (2 months), frozen storage did not significantly increase the cell structure damage in the mangoes. As shown by Li, Zhao, Zhang, Xiao, Sablani, Qu, and Tang [14], mango frozen at −18 °C, which was in its rubbery state, showed only a slight ice crystal growth during the first two months of storage from the mean size of 120 to 136 μm.

### 3.2. Hot Air Drying Kinetics

**Figure 2:**
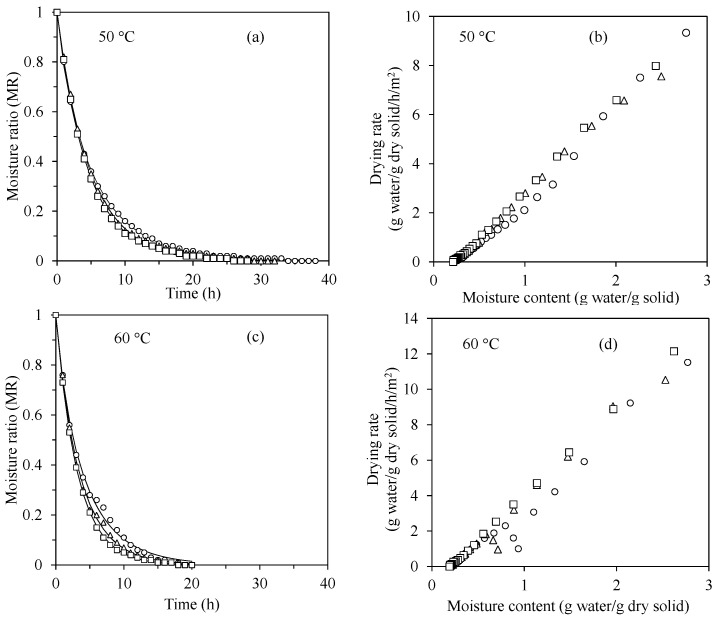
Drying curves (**a**,**c**) and drying rates (**b**,**d**) of (○) fresh, (△) quick frozen, and (□) slow frozen mangoes at 50 and 60 °C in convective hot air dryer. Lines for drying curves were drawn using Page’s model.

The fresh and frozen mangoes (0 month) were dried to evaluate the effect of freezing on drying behavior using a hot air dryer at 50 and 60 °C, which were common drying temperatures for osmotic-dehydrated mangoes. As shown in Figure 2a,c, the typical drying curves were observed for all samples at both drying temperatures, even though the drying curves of the frozen samples showed slight discrepancies from the fresh mangoes. The Page’s model, which is relatively simple and widely used [18], could adequately describe the drying curves with low RSE (0.004–0.018) and very high *R*^2^ values (0.9959–0.9998) (Table 2). Therefore, evaluation of other kinetic models was unnecessary. Comparing the model’s parameters, *k* and *n* revealed that *k* values at 60 °C were significantly higher than those at 50 °C, which can be expected because *k* is usually found to depend on drying temperature [34]. However, comparing the drying curves from different samples at the same drying temperature was not straightforward. At 50 °C, the *k* value for fresh mango was not different from that for slow freezing but higher than the quick-frozen sample, while the lower *n* value of the fresh sample highly contributed to the difference in the observed drying behavior (Figure 2a). On the other hand, at 60 °C, both *k* values for fresh and frozen mangoes were not significantly different, while *n* was also the lowest for the fresh sample. Ando, Okunishi, and Okadome [11] showed that freezing pretreatment at –20 °C for only 4 h resulted in a higher effective diffusion coefficient of moisture at every drying temperature (40, 60, and 80 °C) in pumpkin slices while Vallespir, Cárcel, Marra, Eim, and Simal [12] also reported that the effective diffusion coefficient for frozen beetroot was more than two times higher than raw one for the drying at 40 °C. In this study, the drying times to reach a constant weight, which reflected the overall moisture diffusivity, were longest for the fresh sample while the quick and slow freezing treatment reduced the drying time approximately 16% and 26% at 50 °C and 5% and 16% at 60 °C, respectively. Figure 2b,d show the drying rates during the hot air drying of different samples. It can be seen that only the falling-rate periods were observed without the constant-rate period in all experiments. The falling-rate period of drying indicates that the rate of water removal from the surface by the hot air is higher than the water migration rate from inside to the surface of the sample, and therefore, the overall drying rate continuously declines as the moisture content is decreased [35]. This drying behavior has been reported by other authors in several raw materials, e.g., beetroot [12] and apricot [36]. It should be noted that the effect of freezing on drying behavior usually depends on the type of food. For example, Vallespir, et al. [37] reported that freezing shortened the drying time up to 17%, 27%, and 34% in beetroot, apple, and eggplant, respectively. Because the drying times at 60 °C were about 1.5–2 times shorter than those at 50 °C, the drying temperature of 60 °C was selected to prepare the dried samples for further quality evaluation.

### 3.3. Dried Mango Quality Attributes

#### 3.3.1. Moisture and Water Activity

Mango samples were dried at 60 °C to prepare the final products for quality evaluation (Table 3 and Table 4). The drying times to reach constant weights were 19, 18, and 16 h for the fresh, quick-frozen, and slow-frozen samples, respectively. However, at the same drying time, frozen storage for 1 and 2 months resulted in slightly lower (*p* < 0.05) water activities and moisture contents compared to the 0-month sample. This agreed with the fact that ice crystal growth can occur during frozen storage [14] and therefore slightly increased the cell damage. Nevertheless, all samples showed water activities in the range 0.55–0.61 and moisture content of 18.68–20.68% (w.b.), which were common for osmotic hot air-dried mango [38].

#### 3.3.2. Color, Browning Index, and PPO Activity

Because a sulfiting agent was not used in these samples, discoloration from browning reactions was observed in the dried products (Table 4). The color measurement indicated that while the lightness (L*) and yellowness (b*) were not significantly different among samples, there were slightly lower positive a* values, which represents the redness in the dried mangoes obtained from mangoes after frozen storage. Another indicator for discoloration in this study was the browning index. The fresh mango provided the highest browning index value, which was approximately two times that of frozen samples. Both a* value and browning index indicated that discoloration was more notable in fresh mango than the frozen ones. Browning pigments in the dried mango could have resulted from both enzymatic and non-enzymatic reactions. Analysis of PPO activity in the dried samples revealed that a considerable amount of the enzyme was present in every sample. It was reported that PPO activities in Sindri, SB Chaunsa, and Tommy Atkins mangoes were only partly inactivated after the hot air drying to 11% moisture content [39]. Consistent with the browning index value, the PPO activity in dried mango prepared from the fresh sample (108.95 U/g d.m.) was also significantly higher than in frozen samples (69.66–88.79 U/g d.m.). In addition, it was found that frozen storage for a longer time tended to lower enzyme activity. This was in agreement with the results from Tangtua, Leksawasdi, and Rattanapanone [8], which reported the gradual decrease in PPO activity during the first 4 months of mango cv. Maha Chanok storage at −24 °C.

#### 3.3.3. Total Phenolic Content

Preserving bioactive compounds is important in fruit drying nowadays. Phenolic compounds are abundant in mango and show various activities [40]. It should be noted that the TPC value measured with Folin–Ciocalteu reagent represents not only various phenolic compounds but also an important antioxidant, ascorbic acid (with the reactivity of 0.662 GAE by mass) [41]. The TPC values were slightly different among samples but varied in a narrow range of 2.40–2.71 mg GAE/g d.m. This indicated that TPC did not significantly change during the frozen storage of mangoes for 2 months. The effects of freezing on the degradation of TPC values were different among plants, but the effects were usually much smaller than the hot air-drying step [42].

#### 3.3.4. Textural Properties

Textural properties are important quality indexes for dried mango. Freezing usually results in losses of firmness of fruits because of cell wall rupture [33]. It was shown that frozen storage at −18 °C for 6 months resulted in a significant decrease in the hardness of the frozen mangoes [14]. In this study, instrumental measurement was performed to evaluate the changes in textural properties of the dried mango samples. Although the probe used in this study was a small one, which is not typical for the texture profile analysis (TPA) [23], it was found that textural parameters similar to those in TPA could be derived (Figure S2). Therefore, the obtained textural properties should be valid for the purpose of the treatment comparison in this study. As shown in Table 3, the dried mangoes obtained from frozen samples showed significantly higher hardness values compared to those from the fresh one and the frozen storage time also increased the values (*p* < 0.05). Damaging of the cell membrane after freezing and frozen storage might result in the collapse of cells and consequently a denser structure after drying. The results on hot air drying of fresh and frozen raspberries [43] indicated that the frozen fruits showed higher volume shrinkage after drying, particularly at a low air velocity of 0.5 m/s. However, because the mangoes were osmotically dehydrated in the sugar solution before the hot air drying, it was found that chewiness and gumminess values of the dried products after frozen storage were also significantly higher than those obtained from the fresh samples, while adhesiveness, springiness, and cohesiveness were not significantly different. It was reported that osmotic treatment could improve the appearance and texture of hot air dried fruit compared to using untreated raw material. Barragán-Iglesias, et al. [44] showed that calcium and osmotic treatment (50 °C, 45° Brix, and 150 min) increased the hardness, chewiness, and gumminess of dried papaya and also reduced shrinkage. Although a formal sensory evaluation was not conducted in this study, the authors’ evaluation revealed that the overall texture characteristics of the dried mangoes from frozen storage were acceptable and distinctive as chewier and gummier than the control, which is interesting for further study.

## 4. Conclusions

This study demonstrated that freezing is a feasible method for mango storage before osmotic hot air drying during the off-season. Freezing increased the solid uptake during the osmotic dehydration and shortened the drying time in hot air drying. In addition, freezing and frozen storage retarded the browning reaction and polyphenol oxidase activities. The hardness, chewiness, and gumminess of the dried mango obtained from frozen mangoes were higher than those from fresh mango and also increased at longer frozen storage times, which provided different texture characteristics for the dried mangoes.

## Figures and Tables

**Table 1:** Parameters (*k*_1_ and *k*_2_) and their standard errors (s.e.) from Peleg’s model for describing NWC and NSC in osmotic dehydration of mangoes.

		Fresh	Quick Freezing			Slow Freezing		
			0 mo	1 mo	2 mo	0 mo	1 mo	2 mo
NWC								
	*k*_1_ (s.e.)	5.16 ^b^	16.68 ^a^	11.35 ^a^	12.17 ^a^	15.57 ^a^	12.45 ^a^	10.79 ^a^
		(0.55)	(1.61)	(1.16)	(1.33)	(1.64)	(1.28)	(1.23)
	*k*_2_ (s.e.) ^ns^	2.57	2.51	2.67	2.86	2.60	2.57	2.95
		(0.06)	(0.13)	(0.10)	(0.12)	(0.14)	(0.12)	(0.11)
	NWC_e_ (s.e.) ^ns^	0.61	0.60	0.63	0.65	0.62	0.61	0.66
		(0.01)	(0.03)	(0.02)	(0.03)	(0.03)	(0.03)	(0.02)
	RSE	0.01	0.02	0.01	0.01	0.01	0.01	0.01
	*R* ^2^	0.9902	0.9678	0.9878	0.9810	0.9943	0.9815	0.9805
NSC								
	*k*_1_ (s.e.) ^ns^	1.61	2.16	1.99	2.53	1.73	1.95	2.02
		(0.44)	(0.29)	(0.24)	(0.38)	(0.28)	(0.24)	(0.27)
	*k*_2_ (s.e.)	1.45 ^a^	0.90 ^bc^	0.83 ^c^	1.02 ^b^	0.98 ^b^	0.79 ^c^	0.88 ^bc^
		(0.05)	(0.03)	(0.03)	(0.04)	(0.03)	(0.03)	(0.03)
	NSC_e_ (s.e.)	1.69 ^b^	2.11 ^a^	2.20 ^a^	1.98 ^a^	2.02 ^a^	2.27 ^a^	2.14 ^a^
		(0.06)	(0.07)	(0.08)	(0.08)	(0.06)	(0.09)	(0.07)
	RSE	0.04	0.05	0.04	0.04	0.03	0.05	0.06
	*R* ^2^	0.9497	0.9746	0.9815	0.9785	0.9924	0.9804	0.9637

The values with different superscript letters in a row are significantly different (*p* < 0.05, Holm’s procedure). ns: not significant. RSE: residual standard error. *R*^2^: coefficient of determination.

**Table 2:** Parameters (*k* and *n*) and their standard errors (s.e.) from Page’s model for describing drying behavior of mango slabs at 50 and 60 °C.

Drying Temperature (°C)	Mango	Drying Time (h)	*k* (s.e.)	*n* (s.e.)	RSE	*R* ^2^
50	Fresh	38	0.2459 (0.0055) ^b^	0.88 (0.01) ^c^	0.005	0.9995
	Quick freezing	32	0.2080 (0.0053) ^c^	1.02 (0.01) ^a^	0.005	0.9996
	Slow freezing	28	0.2284 (0.0056) ^b^	0.97 (0.01) ^ab^	0.007	0.9993
60	Fresh	19	0.2983 (0.0068) ^a^	0.88 (0.01) ^c^	0.018	0.9959
	Quick freezing	18	0.3095 (0.0074) ^a^	0.94 (0.01) ^b^	0.013	0.9979
	Slow freezing	16	0.3186 (0.0079) ^a^	0.99 (0.02) ^ab^	0.004	0.9998

The values with different superscript letters in a column are significantly different (*p* < 0.05, Holm’s procedure). RSE: residual standard error. *R*^2^: coefficient of determination.

**Table 3:** Quality attributes of dried mango obtained by hot air drying at 60 °C.

Dried Mango Quality Attributes	Fresh	Quick Freezing			Slow Freezing		
		0 mo	1 mo	2 mo	0 mo	1 mo	2 mo
Drying time (h)	19	18	18	18	16	16	16
Water activity	0.57 ± 0.01 ^b^	0.60 ± 0.01 ^a^	0.58 ± 0.01 ^b^	0.55 ± 0.00 ^c^	0.61 ± 0.01 ^a^	0.58 ± 0.01 ^b^	0.58 ± 0.00 ^b^
Moisture content (% w.b.)	20.68 ± 1.65 ^a^	20.09 ± 1.26 ^ab^	18.68 ± 0.40 ^b^	19.02 ± 1.00 ^ab^	20.55 ± 0.88 ^ab^	18.74 ± 0.73 ^b^	19.87 ± 1.20 ^ab^
Browning index (OD/g d.m.)	0.463 ± 0.03 ^a^	0.266 ± 0.02 ^b^	0.218 ± 0.01 ^c^	0.204 ± 0.01 ^c^	0.218 ± 0.01 ^b^	0.199 ± 0.00 ^c^	0.270 ± 0.03 ^b^
PPO activity (U/g d.m.)	108.95 ± 6.44 ^a^	88.79 ± 5.46 ^b^	69.10 ± 5.66 ^d^	73.55 ± 2.55 ^bcd^	87.61 ± 6.77 ^bc^	71.07 ± 1.23 ^cd^	69.66 ± 4.77 ^d^
TPC (mg GAE/g d.m.)	2.55 ± 0.17 ^abc^	2.71 ± 0.07 ^a^	2.55 ± 0.04 ^abc^	2.47 ± 0.01 ^bc^	2.70 ± 0.00 ^a^	2.63 ± 0.05 ^ab^	2.40 ± 0.02 ^b^
Hardness (g)	146.20 ± 35.15 ^c^	249.89 ± 13.35 ^b^	352.53 ± 14.16 ^a^	371.37 ± 14.70 ^a^	279.34 ± 6.76 ^b^	348.31 ± 10.34 ^a^	352.91 ± 8.07 ^a^
Adhesiveness ^ns^	−15.52 ± 1.48	−17.04 ± 0.41	−27.66 ± 12.97	−36.41 ± 20.16	−17.28 ± 2.32	−28.18 ± 10.86	−18.31 ± 11.53
Springiness ^ns^	0.97 ± 0.07	1.02 ± 0.06	0.98 ± 0.01	1.08 ± 0.07	1.07 ± 0.11	0.97 ± 0.02	1.19 ± 0.19
Cohesiveness ^ns^	0.76 ± 0.02	0.73 ± 0.01	0.66 ± 0.13	0.62 ± 0.16	0.66 ± 0.01	0.65 ± 0.09	0.69 ± 0.14
Chewiness	100.48 ± 12.71 ^b^	177.17 ± 29.08 ^ab^	220.71 ± 135.61 ^ab^	378.02 ± 185.94 ^a^	165.93 ± 20.16 ^b^	198.34 ± 0.48 ^ab^	238.94 ± 19.84 ^ab^
Gumminess (g)	106.95 ± 21.90 ^b^	171.65 ± 15.91 ^ab^	227.49 ± 143.85 ^ab^	352.95 ± 149.30 ^a^	159.44 ± 8.80 ^b^	207.89 ± 0.76 ^ab^	218.95 ± 43.86 ^ab^

Data are expressed as mean ± standard deviation. The values with different superscript letters in a row are significantly different (*p* < 0.05, Duncan’s test). ns: not significant.

**Table 4:** Appearance and color parameters of osmotic hot air-dried mango slabs.

	Fresh	Quick Freezing			Slow Freezing		
		0 mo	1 mo	2 mo	0 mo	1 mo	2 mo
(Peel side)							
L* ^ns^	53.31 ± 3.62	50.96 ± 5.71	54.37 ± 5.50	54.42 ± 5.18	53.76 ± 3.32	54.74 ± 4.74	54.03 ± 4.58
a*	20.04 ± 2.29 ^a^	19.21 ± 2.86 ^a^	15.20 ± 2.79 ^b^	14.81 ± 2.55 ^b^	19.30 ± 1.78 ^a^	15.21 ± 2.36 ^b^	15.03 ± 1.56 ^b^
b* ^ns^	53.35 ± 6.25	49.61 ± 9.15	57.66 ± 6.96	53.01 ± 6.85	58.47 ± 8.99	56.79 ± 5.04	55.35 ± 5.45
ΔE ^ns^	-	9.51 ± 7.88	9.94 ± 4.91	9.02 ± 4.90	9.92 ± 5.81	8.68 ± 3.24	8.26 ± 3.29
	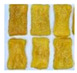	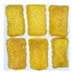	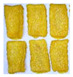	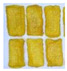	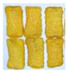	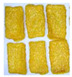	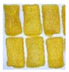
(Inner side)							
L* ^ns^	52.60 ± 2.40	51.64 ± 5.11	54.21 ± 4.07	55.75 ± 4.58	53.10 ± 3.38	53.83 ± 4.29	54.88 ± 3.92
a*	19.19 ± 2.14 ^a^	19.21 ± 2.30 ^a^	17.41 ± 2.49 ^ab^	15.88 ± 2.99 ^b^	18.82 ± 2.17 ^a^	17.09 ± 1.98 ^ab^	16.37 ± 1.61 ^b^
b* ^ns^	50.80 ± 3.67	50.99 ± 9.07	55.34 ± 5.54	53.33 ± 6.52	55.95 ± 6.76	53.70 ± 6.25	55.76 ± 5.52
ΔE ^ns^	-	8.62 ± 5.64	8.58 ± 3.36	8.62 ± 4.95	7.91 ± 4.56	7.95 ± 3.84	8.74 ± 3.94
	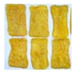	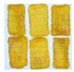	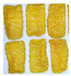	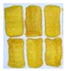	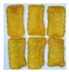	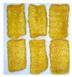	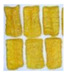

Data are expressed as mean ± standard deviation. The values with different superscript letters in a row are significantly different (*p* < 0.05, Duncan’s test). ns: not significant.

## Data Availability

Research data are available from the corresponding author upon request.

## Supplementary Materials

The following supporting information can be downloaded at https://www.mdpi.com/article/10.3390/foods11030489/s1, Figure S1: Freezing curves of mango slabs in (a) conventional freezer and (b) air-blast freezer, Figure S2: Texture Analyzer graph using p/2 probe, Table S1: Quality indices of fresh mangoes used in this study.
