# Supplementary material for: Osmotic Dehydration, Drying Kinetics, and Quality Attributes of Osmotic Hot Air-Dried Mango as Affected by Initial Frozen Storage
Source: Foods. 2022 Feb 8;11(3):489. doi: 10.3390/foods11030489 (PMC8834474; doi:10.3390/foods11030489)
Supplement: Supplementary file 1 [file foods-11-00489-s001.zip › foods-1573922-supplementary.pdf]

Supplementary information

Osmotic dehydration, drying kinetics, and quality attributes of osmotic hot air-dried mango as affected by initial frozen storage

Pramote Khuwijitjaru[1], Supawadee Somkane[1], Kyuya Nakagawa[2], Busarakorn Mahayothee[1]*

[1]Department of Food Technology, Faculty of Engineering and Industrial Technology, Silpakorn University, Nakhon Pathom 73000, Thailand

[2]Department of Chemical Engineering, Faculty of Engineering, Kyoto University, Katsura, Nishikyo-ku, Kyoto 615-8510, Japan

*Correspondence. E-mail: mahayothee_b@su.ac.th (B. Mahayothee)

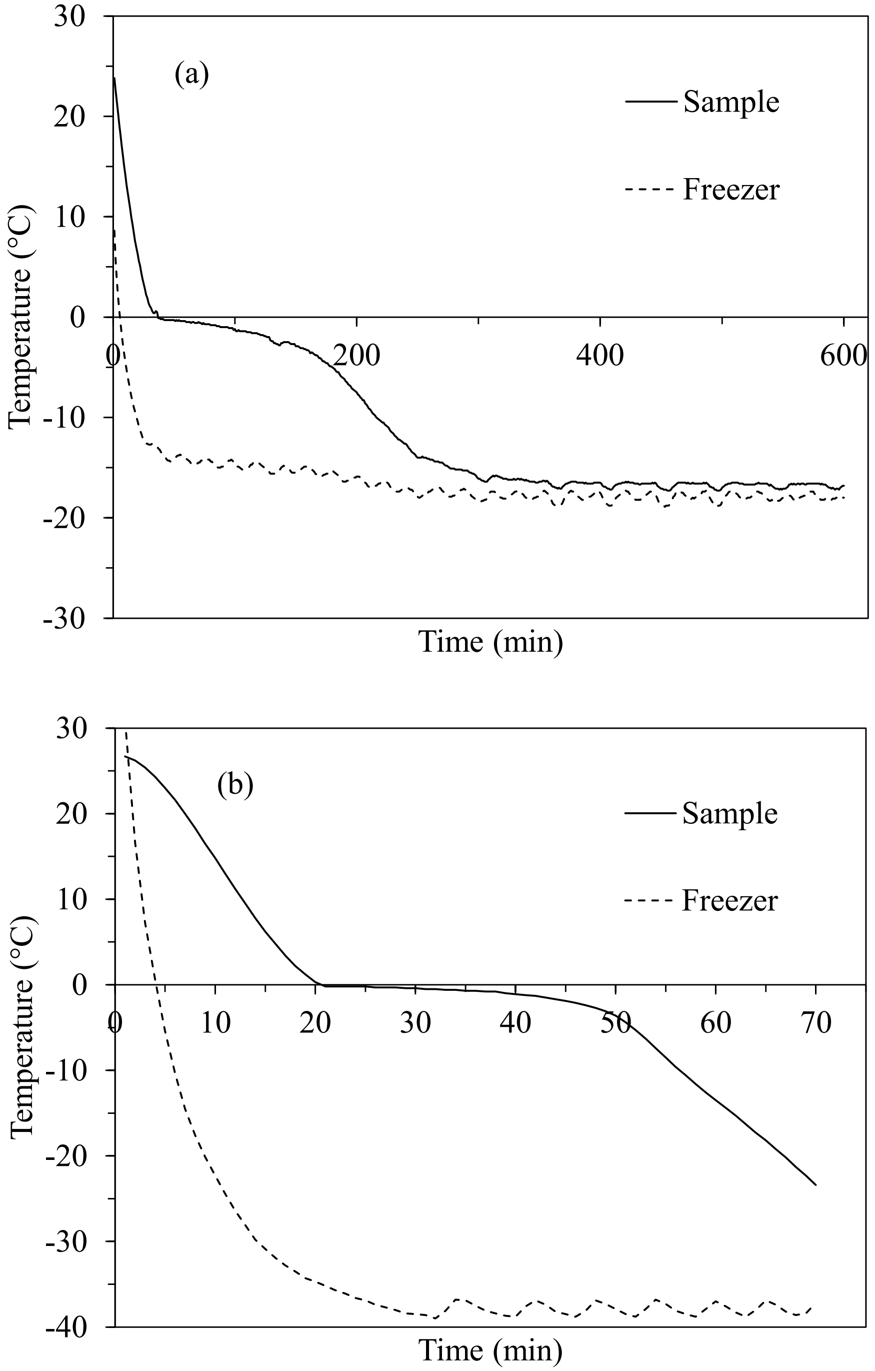

Figure S1. Freezing curves of mango slabs in (a) conventional freezer and (b) air-blast freezer.

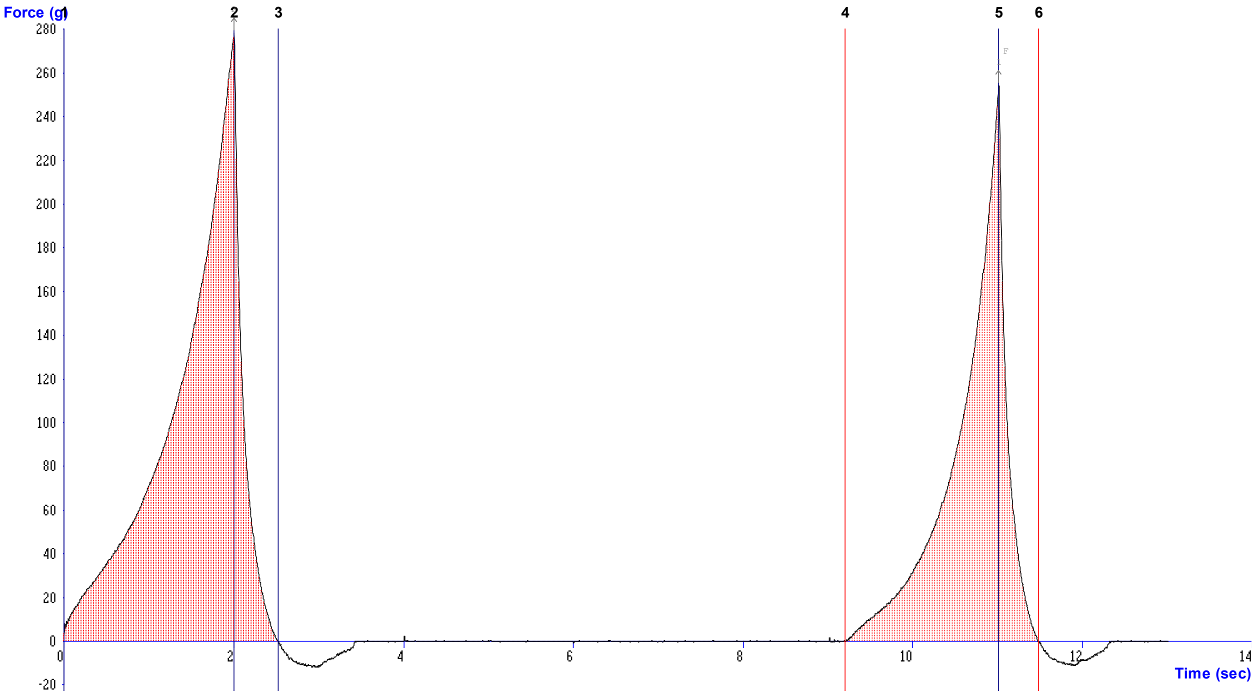

Figure S2. Figure S2. Texture Analyzer graph using p/2 probe.

Table S1. Quality indices of fresh mangoes used in this study.

Quality indices	Range	
Moisture content (% w.b.)	78.89–82.56	
Total soluble solids (°Brix)	18.7–19.2	
Titratable acidity (%)	0.74–1.08	
pH	3.63–4.28	
Hardness (g)	27.53–58.12	
Flesh color	Peel side	Inner side
L*	43.75–61.32	41.57–57.28
a*	11.27–18.09	10.63–18.31
b*	38.18–64.22	34.46–61.07
C*	39.88–66.76	36.07–62.80
h°	41.57–57.59	70.70–77.68
